# Chronic Disease Population Risk Tool (CDPoRT): a study protocol for a prediction model that assesses population-based chronic disease incidence

**DOI:** 10.1186/s41512-018-0042-5

**Published:** 2018-10-01

**Authors:** Ryan Ng, Rinku Sutradhar, Walter P. Wodchis, Laura C. Rosella

**Affiliations:** 1 Dalla Lana School of Public Health, University of Toronto, 155 College St, 6th floor, Toronto, Ontario M5T 3M7 Canada; 2 Institute for Clinical Evaluative Sciences, 2075 Bayview Ave, Toronto, Ontario M4N 3M5 Canada; 3 Institute of Health Policy, Management and Evaluation, University of Toronto, 155 College Street, Toronto, Ontario M5T 3M6 Canada; 4 Institute for Better Health, Trillium Health Partners, 100 Queensway West – Clinical Administrative Building, 6th floor, Mississauga, Ontario L5B 1B8 Canada

**Keywords:** Chronic disease, Prediction model, Prognosis, External validation, Royston-Parmar model, Study protocol

## Abstract

**Background:**

Population-based risk prediction tools exist for individual chronic diseases. From a population health perspective, studying chronic diseases together provides a comprehensive view of the burden of disease in the population. Thus, public health officials and health policymakers would benefit from a prediction tool that measures the incidence of chronic diseases compositely. This study protocol proposes the development and validation of the Chronic Disease Population Risk Tool (CDPoRT) that will predict the incidence of six chronic diseases in the population setting using multivariable modeling techniques.

**Methods:**

CDPoRT will be built using population-based responses to the first six cycles of the Canadian Community Health Survey linked to health administrative data in Ontario and Manitoba from 2000 to 2014. Predictors including modifiable lifestyle risk factors (i.e., alcohol consumption, cigarette smoking, diet, and physical activity) will be used to predict time-to-chronic disease incidence (i.e., congestive heart failure, chronic obstructive pulmonary disease, diabetes, lung cancer, myocardial infarction, and stroke including transient ischemic heart attack). Sex-specific Royston-Parmar models will be used for model development and validation with death free of chronic disease as a competing risk. CDPoRT will be developed using an Ontario derivation cohort consisting of 47,960 females and 38,267 males with 7035 and 6220 chronic disease events, respectively. The model will be validated using split-sample validation using an Ontario validation cohort consisting of 20,325 females and 16,627 males with 2972 and 2658 chronic disease events, respectively. The model will be externally validated in the Manitoba validation cohort (i.e., geographic validation) expected to consist of 11,800 females and 9700 males with 1650 and 1550 chronic disease events, respectively. Measures of overall predictive accuracy (e.g., Nagelkerke’s *R*^2^), discrimination (e.g., Harrell’s concordance statistic), and calibration (e.g., calibration plots) will be used to assess predictive performance.

**Discussion:**

To the extent of our knowledge, CDPoRT will be the first population-based regression prediction model that will predict the incidence of multiple chronic diseases simultaneously at the population level.

**Electronic supplementary material:**

The online version of this article (10.1186/s41512-018-0042-5) contains supplementary material, which is available to authorized users.

## Background

Chronic disease is a global public health issue. In 2014, more than two thirds of deaths worldwide (38 million) were attributed to chronic diseases [1], of which the majority (82%) were attributable to four chronic diseases: cancer, cardiovascular disease, chronic respiratory disease, and diabetes. This burden is mirrored in Canada where 60% of Canadians aged 20 years and older have at least one chronic disease [2]. Chronic disease is responsible for the decreased quality of life and over 200,000 Canadian deaths a year, 27% of which are premature (i.e., deaths under 70 years of age) [1–3].

While the increasing prevalence of chronic disease over time can be attributed to improved chronic disease management [4, 5], one consequence is high costs borne by the health care system due to improved care. In Canada, approximately 42% of direct medical costs and 65% of indirect costs are attributable to chronic disease [6], and individuals with at least one chronic disease are more likely to use the health care system and have higher health expenditures than individuals without chronic disease [7]. The costs are expected to increase as the prevalence of multimorbidity—the presence of two or more chronic conditions—has been increasing in recent years [8]. Multimorbid patients have higher health care expenditures than other patients due to their complexity of care [9–11].

The best strategy to address the chronic disease burden long-term is through prevention [12]. Well-established evidence shows that four modifiable lifestyle risk factors—alcohol consumption, cigarette smoking, unhealthy diet, and physical inactivity—are major risk factors of cancer, cardiovascular disease, chronic respiratory disease, and diabetes and that two thirds of incident cases of these chronic diseases are caused by these risk factors [12]. Despite this knowledge, designing and implementing prevention strategies to reduce chronic disease is difficult. Part of the difficulty is that there is no straightforward way to predict how a prevention strategy targeted towards unhealthy lifestyle behaviors will reduce the incidence of multiple chronic diseases simultaneously. Currently, there are population-based prediction tools that predict the incidence of individual chronic diseases based on lifestyle behaviors [13–16], but to the best of our knowledge, there is no population-based regression prediction model that predicts the incidence of multiple chronic diseases concurrently.

To address the need for a population-based regression prediction model for chronic disease incidence, we propose the development and validation of the Chronic Disease Population Risk Tool (CDPoRT). CDPoRT will use self-reported, modifiable lifestyle risk factor information for a population-based prediction of the incidence of six chronic diseases over a 15-year period: congestive heart failure (CHF), chronic obstructive pulmonary disease (COPD), diabetes, lung cancer, myocardial infarction (MI), and stroke including transient ischemic attack (TIA). A study protocol outlining the analytical details of the development and validation of a prediction model is essential to providing transparency of the model building process and improving the quality of prognostic research [17]. This study protocol outlines the analytical approach that will be used to develop and validate CDPoRT.

## Methods

### Data sources

CDPoRT will use population-based survey data from the Canadian Community Health Survey (CCHS) linked to health administrative data from Ontario and Manitoba, two Canadian provinces. The CCHS is a cross-sectional survey originating in 2000 that collects personal health status, health care utilization, and health determinant data [18]. The CCHS features a multistage, stratified cluster survey design and represents over 98% of the Canadian population 12 years and older. The CCHS will provide the predictors (e.g., modifiable lifestyle risk factors) for CDPoRT.

CCHS respondents will be individually linked to health administrative data in Ontario and Manitoba housed at the Institute for Clinical Evaluative Sciences and Manitoba Centre for Health Policy, respectively. Health administrative data will be used to determine chronic disease outcomes. The data holdings from the Institute for Clinical Evaluative Sciences are hospital discharge data from the Discharge Abstract Database, physician billing claims data from the Ontario Health Insurance Plan Claims Database, cancer registry data from the Ontario Cancer Registry, demographic data from the Registered Persons Database, and cause of death from the vital statistics data, the Office of the Registrar General Deaths Database. From the Manitoba Centre for Health Policy, the data holdings to be used are hospital discharge data from the Discharge Abstract Database, physician claims data from the Medical Services Database, cancer registry data from the Manitoba Cancer Registry, demographic data from the Manitoba Health Insurance Registry, and vital statistics data.

### Study design

Two sex-specific CDPoRT models will be developed and validated, one for females and another for males. There will be one derivation cohort and two validation cohorts. The Ontario derivation cohort consists of 70% of the CCHS respondents from Ontario randomly selected from the first six cycles of the CCHS—cycles 1.1 (2000), 2.1 (2003), 3.1 (2005), 4.1 (2007), 2009/2010, and 2011/2012—that permitted the linkage to Ontario administrative data. The first validation cohort, the Ontario validation cohort, will consist of the other 30% Ontario CCHS respondents. This validation cohort will allow for split-sample validation. The second validation cohort, the Manitoba validation cohort, will be all CCHS respondents from Manitoba who partook in the first six CCHS cycles and permitted the linkage to the Manitoba administrative data. The second validation cohort will enable external, geographic validation. For all cohorts, survey respondents will be excluded if they were under the age of 20 years as of the CCHS interview data or had a self-reported history of the six chronic diseases of interest based on self-report from the CCHS or algorithmic diagnosis from the health administrative data. For individuals who had multiple survey responses, the earliest record after the age of 20 years was used.

### Main predictors—modifiable lifestyle risk factors

The main predictors in CDPoRT are four modifiable lifestyle risk factors: alcohol consumption, cigarette smoking, diet, and physical activity. Information regarding the lifestyle factors was consistently collected across all six CCHS cycles. Alcohol consumption was measured in terms of whether alcohol was consumed in the year prior, the frequency of drinking alcohol, the days on which alcohol was consumed in the last week, and the number of drinks per day. Cigarette smoking was measured in terms of current and former smoking habits including the number of cigarettes smoked daily, the frequency of smoking, and time since quitting smoking. Diet was captured through daily fruit and vegetable consumption based on the daily frequency consumption habits for fruits, fruit juice, starchy vegetables (e.g., potatoes), salad, carrots, and other vegetables. Physical activity was measured by calculating the average metabolic equivalents of daily leisure physical activity for a variety of leisure activities, such as walking, swimming, running, and sports. The modifiable lifestyle risk factor information measured in the CCHS will be used to create a summary predictor for each lifestyle factor (described further in the “Data cleaning and coding of predictors” section). Further details about the data collected by the CCHS for each lifestyle factors can be found in Additional file 1.

### Outcome—chronic disease

Through the linkage to administrative data, individuals will be followed up longitudinally for the incidence of their first chronic disease, defined as CHF, COPD, diabetes, lung cancer, MI, and stroke including TIA. These six chronic diseases were chosen based on a combination of factors, such as their prevalence, previous causal associations with the modifiable lifestyle risk factors (e.g., smoking and lung cancer), impact on morbidity and mortality, and interest from the knowledge users. All selected chronic diseases are readily identifiable from the administrative data. Except for lung cancer, all chronic diseases will be identified from the health administrative data using chronic disease algorithms based on physician diagnosis (Table 1). All chronic disease algorithms have been previously validated in Ontario (sensitivity from 60.2–95.0%, specificity from 76.5–99.2%) [19–24]. For both jurisdictions, the same algorithms will identify chronic diseases based on the International Classification of Diseases (ICD) coding scheme using hospital discharge data (ICD-9 and ICD-10) and physician claims data (ICD-9). Lung cancer will be ascertained from each province’s respective cancer registries [22, 25]. Additionally, vital statistics data will be used to supplement chronic disease incidence by attributing causes of death due to a chronic disease of interest as a diagnosis. This is essential because some chronic diseases would be underreported if only the health services databases were used. For example, some individuals suffer and die from stroke outside of the health care system, so stroke incidence would be underreported if vital statistics were not used. Respondents will be followed up from the date of the CCHS interview up to a maximum of 15 years (January 2000 to December 2014) until one of the following events: incidence of chronic disease, death free of chronic disease (a competing risk), or end of follow-up (December 31, 2014).

**Table 1:** Chronic disease ascertainment algorithms and their predictive accuracy

Chronic disease	Algorithm	Hospital discharge codes		Physician claim codes (ICD-9)	Cancer registry codes (ICD-O-3)	Cause of death (ICD-9)	Sensitivity (95% CI)	Specificity (95% CI)
		ICD-9	ICD-10					
Congestive heart failure (CHF) [19]	2 hospitalization records in a 1-year period *or* 1 physician claim and 1 hospitalization record in a 1-year period *or* 2 physician claim records in a 1-year period *or* death certificate cause of death	428	I50.0, I50.1, I50.9	428	N/A	428	84.8 (77.7, 92.0)	97.0 (96.3, 97.9)
Chronic obstructive pulmonary disease (COPD) [20]	1 hospitalization record *or* 1 physician claim records *or* death certificate cause of death	491, 492, 496	J41, J42, J43, J44	491, 492, 496	N/A	491, 492, 496	85.0 (77.0, 91.0)	78.4 (73.6, 82.7)
Diabetes [21]	1 hospitalization record *or* 2 physician claim records in a 2-year period *or* death certificate cause of death	250	E10, E11, E13, E14	250	N/A	250	86	97
Lung cancer [22]	1 cancer registry record in the Ontario Cancer Registry *or* death certificate cause of death	N/A	N/A	N/A	C34.0, C34.1, C34.2, C34.3, C34.8, C34.9	162.2, 162.3, 162.4, 162.5, 162.8, 162.9	N/A	N/A
Myocardial infarction (MI) [23]	1 hospitalization record *or* death certificate cause of death	410	I21	N/A	N/A	410	95.0	88.0
Stroke including transient ischemic attack (TIA) [24]	1 hospitalization record *or* 2 physician claim records in a 1-year period *or* death certificate cause of death	362.3, 430, 431, 434, 435, 436	G45 (excluding G45.4), H34.0, H34.1, I60, I61, I63 (excluding 163.6), I64	432, 435, 436	N/A	362.3, 430, 431, 434, 435, 436	60.2 (50.7, 69.6)	99.2 (99.0, 99.5)

### Sample size

The Ontario derivation and validation cohorts have already been created but not used for prediction modeling. The female and male derivation cohorts consist of 47,960 and 38,267 respondents with 365,771 and 291,638 years of follow-up, respectively. The minimum sample size required for survival analysis-based prediction is based on the events where the cohort should have at least ten events per degrees of freedom (df) used in the model [26]. There will be 7035 and 6220 chronic disease events for females and males in the derivation cohort, which allows for a maximum of 703 and 622 dfs in the models, respectively. We do not anticipate exceeding 622 dfs during model development.

For validation studies of prognostic models, it has been recommended that the validation cohort has a minimum of 100 events, but ideally 200 (or more) events [27]. The Ontario validation cohort will consist of 20,325 females and 16,627 males, of which 2972 and 2658 had a chronic disease event, respectively. At the time of publication, the Manitoba validation cohort has not been linked to the administrative data, but using the number of CCHS respondents from Manitoba and making assumptions about the proportion of individuals who consented to the linkage and did not meet the exclusion criteria based on the Ontario cohorts, the Manitoba validation cohort is expected to consist of 11,900 females and 9600 males. Using the sex-specific incidence of chronic disease in the Ontario cohorts, approximately 1800 females and 1600 males are expected to have chronic disease events in the Manitoba validation cohort. Both validation cohorts exceed the recommended number of events for validation.

### Analysis plan

The analysis plan was developed with consideration of the guidelines provided by Harrell and Steyerberg [28, 29]. To protect against type 1 error from a data-driven variable selection of model specification, the predictor-outcome associations in any cohort will not be explored until the protocol is published. All steps in the analytical plan for the development and validation of CDPoRT are guided by the principle of creating a prediction model with high predictive accuracy (e.g., calibration, discrimination). The analytical plan also takes into consideration that CDPoRT will be used by knowledge users (e.g., public health units, health policymakers, and other decision-makers) that may not have extensive statistics training, so the practical application of CDPoRT is important. Features of CDPoRT that will make it more user-friendly include easy input of the predictors, straightforward and meaningful interpretations of the predictions overall and by subgroups, and consistent model application across time and geography. As a result, the practical considerations will impact how CDPoRT is built and will affect the aspects of model development, such as predictor selection and operationalization, missing data management, model specification, model estimation, validation, and model presentation. All data will be cleaned and manipulated using SAS. Modeling will be conducted using Harrell’s *Hmisc* package of function in R and the *stpm2* command in Stata. The TRIPOD statement for multivariable predictive models also helped guide this study protocol and will be used to help report the estimates from CDPoRT [30].

#### Identification of predictors

The modifiable lifestyle risk factors were identified based on well-established evidence of their associations with the chronic diseases of interest [1, 31–33]. Other candidate predictors were identified based on a combination of subject matter expertise, input from the knowledge users, the group’s previous experiences with population-based prediction models [13–16, 34–36], and predictor availability across all CCHS cycles. During this phase, some predictors were excluded due to narrow distributions or insufficient variation, while others were excluded based on redundancy. There were sixteen candidate predictors identified: four modifiable lifestyle risk factors (alcohol consumption, cigarette smoking, daily fruit and vegetable consumption, physical activity), six sociodemographic characteristics (age, ethnicity, immigration status, household income, education, marital status), and six other health-related factors (asthma, body mass index (BMI), high blood pressure, household secondhand smoke, self-rated health, life stress). Self-reported BMI is known to be biased, so a validated correction equation will be used [37]. Because the biological underpinnings of chronic disease vary by sex, two CDPoRT models will be created.

#### Data cleaning and coding of predictors

Continuous predictors will be examined using descriptive statistics, histograms, and box plots to examine their distributions. Incorrect values will either be corrected or set to missing. Continuous predictors with highly skewed distributions will be truncated to the 99.5th percentile. Continuous predictors that can be meaningfully categorized will be grouped; for example, BMI will be classified according to internationally recognized groups (i.e., underweight, normal weight, overweight, and classes I to III obesity). The frequency distribution of categorical predictors will be examined, and categories which are too small (i.e., < 5%) will be grouped with another category. Based on previous experiences, we will be deriving some predictors based on a combination of survey responses. For example, alcohol consumption status will be defined based on whether the person reported drinking in the past year, the number of times drank in the past week, and the total number of drinks consumed while considering sex-specific differences (i.e., non-drinker, light drinker, moderate drinker, heavy drinker, binge drinker). Predictors will also be created with consideration for how they were defined in similar population-based prediction models [13–16, 34–36]. The definitions for all predictors have been pre-specified to minimize overfitting (Table 2). However, we recognize that there is some subjectivity in how these classifications are made, so sensitivity analyses of the predictive performance of other definitions of the predictors will be performed to examine the robustness of our definitions during model building and validation.

**Table 2:** Pre-specified definitions of CDPoRT predictors

Variable type	Variable	Definition	df
Modifiable lifestyle risk factors	Alcohol consumption		4
	Non-drinker	No alcohol consumption in the last 12 months or drink frequency fewer than once a week	
	Light drinker	Alcohol consumption frequency at least once a week and 0–2 (females) or 0–3 (males) drinks in the previous week	
	Moderate drinker	3–14 (females) or 4–21 (males) drinks in the previous week	
	Heavy drinker	≥ 14 (females) or ≥ 21 (males) drinks in the previous week, or binging behavior on a weekly basis (≥ 5 drinks on any occasion)	
	Cigarette smoking		5
	Non-smoker	Never a smoker or former occasional smoker with < 100 lifetime cigarettes	
	Heavy smoker	Current smoker [≥ 1 pack (25 cigarettes)/day]	
	Light smoker	Current smoker [< 1 pack (25 cigarettes)/day]	
	Former heavy	Former smoker [≥ 1 pack (25 cigarettes)/day]	
	Former light smoker	Former smoker [< 1 pack (25 cigarettes)/day]	
	Daily fruit and vegetable consumption		3
	Low consumption	0 to < 3 times daily	
	Medium consumption	3 to < 6 times daily	
	High consumption	≥ 6 times daily	
	Physical activity quartile		4
	Quartile 1	Bottom 25% physically active	
	Quartile 2	Bottom 26–50% physically active	
	Quartile 3	Bottom 51–75% physically active	
	Quartile 4	Top 25% physically active	
Sociodemographic characteristics	Age		5
	Continuous	Spline with 4 interior knots	
	Ethnicity		2
	White		
	Non-White		
	Immigration status		3
	Canadian born		
	Recent immigrant	Immigrated < 10 years	
	Non-recent immigrant	Immigrated ≥ 10 years	
	Household income		5
	Quintile 1	Lowest 20%	
	Quintile 2		
	Quintile 3		
	Quintile 4		
	Quintile 5	Highest 20%	
	Education		3
	Less than secondary school graduation		
	Secondary school graduation		
	Post-secondary education (complete and partial)		
	Marital status		3
	Single never married		
	Domestic partner (married/common law)		
	Widowed/separated/divorced		
	Asthma		2
	Yes		
	No		
	Body mass index (BMI) (kg/m2)		6
	Underweight	< 18.5 BMI	
	Normal weight	18.5–24.9 BMI	
	Overweight	25.0–29.9 BMI	
	Moderately obese (class 1)	30.0–34.9 BMI	
	Very obese (class 2)	35.0–39.9 BMI	
	Severely obese (class 3)	≥ 40.0 BMI	
	High blood pressure		2
	Yes		
	No		
	Household secondhand smoke		2
	Household secondhand smoke		
	No household secondhand smoke		
	Self-rated health		3
	Excellent/very good		
	Good		
	Fair/poor		
	Life stress		4
	Quite a bit/extremely stressful		
	Not very stressful		
	A bit stressful		
	Not at all stressful		

The degrees of freedom (df) include a category for those who could not be classified (i.e., missing)

#### Missing data

Variables with missing data will be categorized as a separate category. This will allow users of the tool to include all respondents during CDPoRT application. While defining a missing category may introduce some classification bias, excluding incomplete cases will add bias by making the cohort less representative of the population. We expect minimal bias from creating a missing category because most predictors have less than 1% of values missing. Income is expected to be an important predictor for chronic disease incidence, so imputed values of income will be used in the cycles in which Statistics Canada provided computed income values.

#### Model estimation

The initial models will be estimated using the Royston-Parmar model [38]. The distinguishing feature of this model is that the baseline cumulative hazard function is modeled as a restricted cubic spline which permits estimation of absolute measures of effect (e.g., cumulative incidence) at all time points. While the Royston-Parmar model is generally robust to the number and placement of knots [38–40], it is recommended to test the robustness of the baseline function by varying the number and placement of knots [41]. To do this, different baseline functions with a varying number of knots (between two and six knots) evenly distributed over the survival times will be compared via information criterion (i.e., Akaike information criterion (AIC), Bayesian information criterion (BIC)). If there is a baseline function with a clear best model fit, that function will be used. If the model fit is similar between multiple baseline functions, the function with the fewest number of knots will be selected to reduce overfitting that may occur during validation. The knot placement of the selected function will then be varied randomly to further test the robustness. The baseline cumulative hazard function will be modeled on the proportional hazards (PH) scale as hazards ratios are a common estimate for survival models. However, the Royston-Parmar model can also be modeled on the proportional odds or probit scale, and if the model fit is greatly improved on either of these scales, that scale will be selected instead.

The Royston-Parmar model estimates the cause-specific hazards ratio, but it can also account for competing risks by transforming the cause-specific hazards to a cumulative incidence function [42]. CDPoRT will consider death free of chronic disease as a competing risk. PH models assume that the relative hazard of a predictor is constant across time. However, this might not be true, and so violations of the PH model will be examined by plotting raw and smoothed scaled Schoenfeld residuals versus time versus predictors that are expected to vary with time (e.g., age). Any violations of the PH assumption that alter the model fit or predictive performance will be accounted for by including an interaction with time. The degree of overfitting will be estimated using the heuristic shrinkage estimator, which is based on the log-likelihood ratio *χ*^2^ statistic of the full model [43]. If the shrinkage is less than 0.9, the model will be adjusted for overfitting accordingly. If the shrinkage value is greater than 0.9, and the model does not perform well, data reduction techniques will be explored. All results will incorporate survey weights so that CDPoRT is representative of the underlying population. Variance estimates will be calculated using the bootstrap survey weights via balanced repeated replication [44, 45].

#### Model specification

Sex-specific models will be fitted initially with the pre-specified forms of the predictors (Table 2). Predictors that were originally continuous will also be modeled in a flexible fashion using restricted cubic splines with the knots placed evenly through the distribution. If the fit is greatly improved with the continuous form of the predictor versus the categorical form based on information criterion (e.g., AIC, BIC), and measures of predictive performance (i.e., overall fit, discrimination, calibration), then the continuous, centered form will be used. While we have pre-specified definitions of the categorical predictors to use, other definitions will be examined during model building. If any of these definitions greatly improve the model fit or predictive performance of the model, the alternate specification will be used instead. Ordinal predictors will be initially modeled as categorical, but if the fit improves when specified as a linear function, the ordinal variable will be modeled continuously. Collinearity between predictors will be assessed using the *Varclus* function in R. Two-way interactions between variables will be examined. The model that uses all the pre-specified forms of the predictors has 56 dfs (Table 2).

There will be a stepwise selection process for predictors. The initial model will consist of the modifiable lifestyle risk factors. The overall fit of the initial model will be analyzed in terms of model fit statistics (e.g., log-likelihood, AIC, BIC). The predictive performance of this initial model will be assessed using overall measures of predictive accuracy, calibration, and discrimination (described further in the “Assessment of predictive performance” section). Two sets of predictors (sociodemographic characteristics, other health-related factors) will be added to the model, one set at a time. Individual predictors within each set that do not improve the overall model fit (e.g., AIC, BIC) and/or predictive performance (e.g., discrimination, calibration) will be removed. When a set of predictors is added to the model, the predictive performance of the existing predictors will be examined, and if their predictive performance is minimal, the predictor may be removed. Variables excluded in prior rounds will be added back to the model to confirm whether their exclusion was appropriate. The overall fit and predictive performance after each set of predictors are added will be compared to the original model. The final model will consist of the four modifiable lifestyle risk factors and a combination of sociodemographic characteristics and other health-related factors.

#### Assessment of predictive performance

Predictive performance in the derivation and validation cohorts will be assessed and reported using overall measures of predictive accuracy, discrimination, and calibration. Accuracy will be measured with Nagelkerke’s *R*^2^ and Brier score. Discrimination will be assessed with Harrell’s concordance statistic. Calibration is important for prediction models because risk prediction in future settings is of primary interest [29]. Calibration will be assessed by comparing the observed and predicted risk of a chronic disease over deciles of risk using calibration plots at specific time points (e.g., 1, 5, 10, and 15 years). Calibration slopes will be generated by regressing the outcome in the validation cohort on the predicted chronic disease risk. Deviation from perfect calibration (i.e., slope of 1) will be tested with Wald or likelihood ratio tests. Calibration-in-the-small will also be assessed for subgroups of interest (e.g., cigarette smoking groups). We consider any relative difference of less than 20% between observed and predicted risk within subgroups that have at least a 5% chronic disease event rate as adequately calibrated.

#### Model validation

Once the model has been developed in the Ontario derivation cohort, the performance of the model will be validated within the Ontario context in terms of overall measures of predictive accuracy, discrimination, and calibration. This will provide an idea of the model’s optimism when validated in the Manitoba context. We will perform bootstrap validation within the Ontario derivation cohort to get an idea of how the model will perform in the Ontario validation cohort. We will also validate the model using the Ontario validation cohort via split-sample validation. The major drawback of split-sample validation is not expected to affect the validation as the sample sizes for the Ontario derivation and validation cohorts are large. While the Ontario cohort is being divided into parts for the split-sample validation, the final regression coefficients using the full Ontario dataset will be used to maximize the sample size and follow-up duration. The final combined model will maintain the same predictors and form as the derivation model. In the off chance that the derivation and validation cohort differ significantly, a cohort-specific intercept and/or interaction term will be included in the Manitoba model.

CDPoRT will undergo external validation using the Manitoba validation cohort. This will assess geographic validation. The performance of the model in the Manitoba validation cohort will be understood using predictive accuracy, discrimination, and calibration. As well, novel approaches to assess geographic validity will be used [46, 47]. Ideally, the CDPoRT model developed and validated in Ontario will have high predictive performance in Manitoba. However, the case-mix of individuals and their outcomes will be different between the jurisdictions, which may result in decreased model performance. In this scenario, CDPoRT in the Manitoba setting will be modified using updating methods (e.g., re-calibration, model revision, or model extension).

#### Model presentation

The final regression model of the Ontario CDPoRT consisting of the derivation and validation sample, as well as the Manitoba CDPoRT, will be presented using estimated hazards ratios and 95% confidence intervals. Absolute measures of effect such as baseline risk and cumulative incidence will also be presented, which is a feature of the Royston-Parmar model versus the Cox PH model because the baseline cumulative hazard function is modeled. Estimation of the baseline risk allows and permits the calculation of the attributable risk of the predictors based on the hazard ratios. Interactive, visual tools will also be created to help describe the model, which can improve the literacy of the model for non-technical audiences. The regression formula will also be published and used as the underpinning for web-based implementation.

#### Analyses beyond initial model development

Sensitivity analyses will be conducted to see how the model performs under different settings. One sensitivity analysis will be performed to see the algorithm’s performance in a population in which individuals were excluded based on self-reported chronic disease only and not based on an algorithmic diagnosis from the health administrative data. This is important to understand how the model will perform in real-world environments where users will not have access to health administrative data. A second sensitivity analysis will be performed where each chronic disease will be modeled separately to see if modeling each chronic disease as separate outcomes and combining results to get the overall chronic disease incidence improves predictive performance. This is a possibility as the predictor coefficients can be thought of as an averaged effect that reflects the frequency of each chronic disease. In a population with a different case-mix of chronic disease, the model may have worse predictive performance. As other chronic diseases (/conditions) remain of interest to the knowledge user [8], a third sensitivity analysis will see how the predictiveness of CDPoRT changes when other chronic conditions are included as outcomes.

## Discussion

Chronic diseases are a public health priority and a great expenditure burden on the Canadian health care system. Accurate prediction of chronic disease incidence in the population based on modifiable lifestyle risk factors will help with the prevention strategies and health care delivery planning. However, public health officials and health policymakers do not have a straightforward way to estimate chronic disease incidence accurately for their jurisdiction. The development and validation of CDPoRT hopes to address these needs.

### Limitations

One limitation of CDPoRT is that while the tool will be representative of most of the population (98%), some groups will not be covered, notably on-reserve Aboriginals. This is an important consideration because Aboriginals have been reported to be at greater risk for developing chronic disease [48], and a sizable proportion of the Manitoba population is Aboriginal (15%) [49]. The Aboriginal population is relatively smaller in Ontario (2%), so it should not impact the model development. However, the different case-mix is expected to impact the validation in the Manitoba context. To help with this matter, we will compare the case-mix between Ontario and Manitoba and use the information to understand how it will affect the calibration and discrimination [29]. Novel approaches to geographic validation will also be explored [46, 47]. If necessary, CDPoRT will be updated to the Manitoba setting.

A second limitation is that the measurement quality of the modifiable lifestyle risk factors varies. Cigarette smoking is the most complete measure as it considers current and past smoking history. However, pack-years smoked cannot be measured because not enough details about the smoking habits of former smokers were collected. Alcohol consumption measures drinking habits up to 1 year from the interview date but does not measure habits before this period. Diet is only captured by fruit and vegetable consumption, and dietary habits of other food groups (e.g., meats, dairy) or constituents (e.g., sodium, fat) are not captured. Specific cycles of the CCHS capture dietary habits in more details (e.g., CCHS 2.2), and if the data can be obtained, reclassification methods can be used to measure the incremental changes in the prediction if these predictors were added to the model [50]. Physical activity only measures leisure physical activity, and other forms of physical activity (e.g., transportation, work) are not measured.

### Implications

CDPoRT will be developed to ensure the tool meets the needs of the knowledge user. Partnerships exist with knowledge users at the municipal and provincial level including prominent health system decision-makers (e.g., Medical Officers of Health; program managers, directors and executives in two provincial governments). This will enable CDPoRT to be applied in various regions and settings across Canada through the support of knowledge brokers and permit knowledge users to predict the incidence of multiple chronic diseases simultaneously in their population. The effectiveness of CDPoRT in these settings will be further evaluated to understand the utility of the tool in real-world settings for supporting decision-making and planning.

### Conclusions

To the extent of our knowledge, CDPoRT will be the first population-based regression prediction model that will predict the incidence of multiple chronic diseases simultaneously at the population level. The tool will be used by public health and health policymakers to support planning and decision-making.

## Additional file

Additional file 1: Modifiable lifestyle risk factors as measured from the Canadian Community Health Survey. (DOCX 49 kb)

## Abbreviations

AIC: Akaike information criterion
BIC: Bayesian information criterion
BMI: Body mass index
CCHS: Canadian Community Health Survey
CDPoRT: Chronic Disease Population Risk Tool
CHF: Congestive heart failure
COPD: Chronic obstructive pulmonary disease
df: Degrees of freedom
ICD: International Classification of Diseases
MI: Myocardial infarction
PH: Proportional hazards
TIA: Transient ischemic attack

## References

[1] World Health Organization (2014). Global status report on noncommunicable diseases 2014.

[2] Betancourt MT, Roberts KC, Bennett T-L, Driscoll ER, Jayaraman G, Pelletier L (2014). Monitoring chronic diseases in Canada: the Chronic Disease Indicator Framework. Chronic Dis Inj Can.

[3] Norheim OF, Jha P, Admasu K (2015). Avoiding 40% of the premature deaths in each country, 2010–30: review of national mortality trends to help quantify the UN Sustainable Development Goal for health. Lancet.

[4] Bodenheimer T, Wagner EHE, Grumbach K (2002). Improving primary care for patients with chronic illness. JAMA.

[5] Coleman K, Austin BT, Brach C, Wagner EH (2009). Evidence on the chronic care model in the new millennium. Health Aff.

[6] Mirolla M, Chronic Disease Prevention Alliance of Canada. The cost of chronic disease in Canada. http://www.gpiatlantic.org/pdf/health/chroniccanada.pdf.

[7] Rosella LC, Fitzpatrick T, Wodchis WP, Calzavara A, Manson H, Goel V (2014). High-cost health care users in Ontario, Canada: demographic, socio-economic, and health status characteristics. BMC Health Serv Res.

[8] Pefoyo AJK, Bronskill SE, Gruneir A (2015). The increasing burden and complexity of multimorbidity. BMC Public Health.

[9] Wodchis WP (2016). Performance measurement for people with multimorbidity and complex health needs. Healthc Q.

[10] Gijsen R, Hoeymans N, Schellevis FG, Ruwaard D, Satariano WA, Van Den BGAM (2001). Causes and consequences of comorbidity. A review.

[11] Thavorn K, Maxwell CJ, Gruneir A (2017). Effect of socio-demographic factors on the association between multimorbidity and healthcare costs: a population-based, retrospective cohort study. BMJ Open.

[12] Beaglehole R, Bonita R, Horton R (2011). Priority actions for the non-communicable disease crisis. Lancet.

[13] Rosella LC, Manuel DG, Burchill C, Stukel TA (2011). A population-based risk algorithm for the development of diabetes: development and validation of the Diabetes Population Risk Tool (DPoRT). J Epidemiol Community Health.

[14] Taljaard M, Tuna M, Bennett C (2014). Cardiovascular Disease Population Risk Tool (CVDPoRT): predictive algorithm for assessing CVD risk in the community setting. A study protocol. BMJ Open.

[15] Fisher S, Hsu A, Mojaverian N, et al. Dementia Population Risk Tool (DemPoRT): study protocol for a predictive algorithm assessing dementia risk in the community. BMJ Open. 2017;7 10.1136/bmjopen-2017-018018. 10.1136/bmjopen-2017-018018 PMC5665213 29070641

[16] Manuel DG, Tuna M, Perez R (2015). Predicting stroke risk based on health behaviours: development of the Stroke Population Risk Tool (SPoRT). PLoS One.

[17] Peat G, Riley RD, Croft P, et al. Improving the transparency of prognosis research: the role of reporting, data sharing, registration, and protocols. PLoS Med. 2014;11 10.1371/journal.pmed.1001671. 10.1371/journal.pmed.1001671 PMC4086727 25003600

[18] Béland Y (2002). Canadian community health survey--methodological overview. Heal reports.

[19] Schultz SE, Rothwell DM, Chen Z, Tu K (2013). Identifying cases of congestive heart failure from administrative data: a validation study using primary care patient records. Chronic Dis Inj Can.

[20] Gershon AS, Wang C, Guan J, Vasilevska-Ristovska J, Cicutto L, To T (2009). Identifying individuals with physician diagnosed COPD in health administrative databases. COPD.

[21] Hux JE, Ivis F, Flintoft V, Bica A (2002). Diabetes in Ontario: determination of prevalence and incidence using a validated administrative data algorithm. Diabetes Care.

[22] McLaughlin JR, Kreiger N, Marrett LD, Holowaty EJ (1991). Cancer incidence registration and trends in Ontario. Eur J Cancer.

[23] Austin PC, Daly PA, Tu JV (2002). A multicenter study of the coding accuracy of hospital discharge administrative data for patients admitted to cardiac care units in Ontario. Am Heart J.

[24] Tu K, Wang M, Young J (2013). Validity of administrative data for identifying patients who have had a stroke or transient ischemic attack using EMRALD as a reference standard. Can J Cardiol.

[25] Lix L, Stat P, Smith M (2016). Cancer data linkage in Manitoba: expanding the infrastructure.

[26] Peduzzi P, Concato J, Feinstein AR, Holford TR (1995). Importance of events per independent variable in proportional hazards regression analysis II. Accuracy and precision of regression estimates. J Clin Epidemiol.

[27] Collins GS, Ogundimu EO, Altman DG (2016). Sample size considerations for the external validation of a multivariable prognostic model: a resampling study. Stat Med.

[28] Harrell FE (2015). Regression modeling strategies.

[29] Steyerberg EW (2009). Clinical prediction models.

[30] Collins GS, Reitsma JB, Altman DG, Moons KGM (2015). Transparent reporting of a multivariable prediction model for Individual Prognosis or Diagnosis (TRIPOD): the TRIPOD statement. Ann Intern Med.

[31] Public Health Agency of Canada (2016). How healthy are Canadians: a trend analysis of Canadians from a healthy living and chronic disease perspective.

[32] Busse R, Blumel M, Scheller-Kreinsen D, Zentner A. Tackling chronic disease in Europe: strategies, interventions, and challenges. Geneva: World Health Organization; 2010. http://www.euro.who.int/__data/assets/pdf_file/0008/96632/E93736.pdf.

[33] Bauer UE, Briss PA, Goodman RA, Bowman BA (2014). Prevention of chronic disease in the 21st century: elimination of the leading preventable causes of premature death and disability in the USA. Lancet.

[34] Manuel DG, Perez R, Sanmartin C (2016). Measuring burden of unhealthy behaviours using a multivariable predictive approach: life expectancy lost in Canada attributable to smoking, alcohol, physical inactivity, and diet. PLoS Med.

[35] Rosella LC, Kornas K, Yao Z (2017). Predicting high health care resource utilization in a single-payer public health care system: development and validation of the High Resource User Population Risk Tool (HRUPoRT). Med Care.

[36] Lebenbaum M, Espin-garcia O, Li Y, Rosella LC (2018). Development and validation of a population based risk algorithm for obesity: the Obesity Population Risk Tool (OPoRT). PLoS One.

[37] Shields M, Gorber SC, Janssen I, Tremblay MS (2011). Bias in self-reported estimates of obesity in Canadian health surveys: an update on correction equations for adults. Health Rep.

[38] Royston P, Parmar MKB (2002). Flexible parametric proportional-hazards and proportional-odds models for censored survival data, with application to prognostic modelling and estimation of treatment effects. Stat Med.

[39] Lambert PC, Royston P (2009). Further development of flexible parametric models for survival analysis. Stata J.

[40] Nelson CP, Lambert PC, Squire IB, Jones DR (2007). Flexible parametric models for relative survival, with application in coronary heart disease. Stat Med.

[41] Ng R, Kornas K, Sutradhar R, Wodchis WP, Rosella LC (2018). The current application of the Royston-Parmar model for prognostic modeling in health research: a scoping review. Diagnostic Progn Res.

[42] Hinchliffe SR, Lambert PC (2013). Flexible parametric modelling of cause-specific hazards to estimate cumulative incidence functions. BMC Med Res Methodol.

[43] Van Houwelingen JC, Le CS (1990). Predictive value of statistical models. Stat Med.

[44] Kovacevic MS, Mach L, Roberts G. Bootstrap variance estimation for predicted individual and population-average risks. Am Stat Assoc. 2008:2289–96. https://www.researchgate.net/publication/264878924_Bootstrap_Variance_Estimation_for_Predicted_Individual_and_Population-Average_Risks.

[45] Yeo D, Mantel H, Liu TP. Bootstrap variance estimation for the national population health survey. Proc Surv Res Methods Sect Am Stat Assoc 1999.

[46] Austin PC, van Klaveren D, Vergouwe Y, Nieboer D, Lee DS, Steyerberg EW (2016). Geographic and temporal validity of prediction models: different approaches were useful to examine model performance. J Clin Epidemiol.

[47] Austin PC, van Klaveren D, Vergouwe Y, Nieboer D, Lee DS, Steyerberg EW (2017). Validation of prediction models: examining temporal and geographic stability of baseline risk and estimated covariate effects. Diagnostic Progn Res.

[48] Bruce SG, Riediger ND, Lix LM (2014). Chronic disease and chronic disease risk factors among First Nations, Inuit and Métis populations of northern Canada. Chronic Dis Inj Can.

[49] Statistics Canada. Aboriginal identity population by age groups, median age and sex, 2006 counts for both sexes, for Canada, provinces and territories - 20% sample data. 2009. http://www12.statcan.gc.ca/census-recensement/2006/dp-pd/hlt/97-558/pages/page.cfm?Lang=E&Geo=PR&Code=01&Table=1&Data=Count&Sex=1&Age=1&StartRec=1&Sort=2&Display=Page. Accessed 28 Mar 2016.

[50] Steyerberg EW, Vickers AJ, Cook NR (2010). Assessing the performance of prediction models: a framework for traditional and novel measures. Epidemiology.

