# Supplementary material for: Chronic Disease Population Risk Tool (CDPoRT): a study protocol for a prediction model that assesses population-based chronic disease incidence
Source: Diagn Progn Res. 2018 Oct 1;2:19. doi: 10.1186/s41512-018-0042-5 (PMC6460781; doi:10.1186/s41512-018-0042-5)
Supplement: Supplementary file 1 — Modifiable lifestyle risk factors as measured from the Canadian Community Health Survey. (DOCX 49 kb) [file 41512_2018_42_MOESM1_ESM.docx]

## Additional file

## Additional file 1. Modifiable lifestyle risk factors as measured from the Canadian Community Health Survey

| **Risk factor** | **Questions** | **Responses** |
| --- | --- | --- |
| Alcohol consumption | During the past 12 months, that is, from [date one year ago] to yesterday, have you had a drink of beer, wine, liquor or any other alcoholic beverage? | Yes, No |
|  | During the past 12 months, how often did you drink alcoholic beverages? | Less than once a month, Once a month, 2 to 3 times a month, Once a week, 2 to 3 times a week, 4 to 6 times a week, Every day |
|  | How often in the past 12 months have you had 5 or more drinks on one occasion? | Never, Less than once a month, Once a month, 2 to 3 times a month, Once a week, More than once a week |
|  | Thinking back over the past week, that is, from [date last week] to yesterday, did you have a drink of beer, wine, liquor or any other alcoholic beverage? | Yes, No |
|  | Starting with yesterday, how many drinks did you have on [Sunday/Monday/Tuesday/Wednesday/Thursday/Friday/Saturday]? | 0 to 99 |
| Cigarette smoking | In your lifetime, have you smoked a total of 100 or more cigarettes (about 4 packs)? | Yes, No |
|  | Have you ever smoked a whole cigarette? | Yes, No |
|  | At the present time, do you smoke cigarettes daily, occasionally or not at all? | Daily, Occasionally, Not at all |
|  | How many cigarettes [do/does] [you/he/she] smoke each day now? | 1 to 99 |
|  | On the days that [you/FNAME] [do/does] smoke, how many cigarettes [do/does] [you/he/she] usually smoke? | 1 to 99 |
|  | In the past month, on how many days [have/has] [you/he/she] smoked 1 or more cigarettes? | 0 to 30 |
|  | [Have/Has] [you/he/she] ever smoked cigarettes daily? | Yes, No |
|  | When did you stop smoking? Was it: | Less than one year ago? 1 year to less than 2 years ago? 2 years to less than 3 years ago? 3 or more years ago? |
|  | How many cigarettes did [you/he/she] usually smoke each day? | 1 to 99 |
|  | When did [you/he/she] stop smoking daily? Was it: | Less than one year ago? 1 year to less than 2 years ago? 2 years to less than 3 years ago? 3 or more years ago? |
|  | Was that when [you/he/she] completely quit smoking? | Yes, No |
|  | When did [you/he/she] stop smoking completely? Was it: | Less than one year ago? 1 year to less than 2 years ago? 2 years to less than 3 years ago? 3 or more years ago? |
| Daily fruit and vegetable consumption | How often do you usually drink fruit juices such as orange, grapefruit or tomato? | 0 to 99 [per day/per week/per month/per year], Never |
|  | Not counting juice, how often do you usually eat fruit? | 0 to 99 [per day/per week/per month/per year], Never |
|  | How often do you (usually) eat green salad? | 0 to 99 [per day/per week/per month/per year], Never |
|  | How often do you usually eat potatoes, not including French fries, fried potatoes, or potato chips? | 0 to 99 [per day/per week/per month/per year], Never |
|  | How often do you (usually) eat carrots? | 0 to 99 [per day/per week/per month/per year], Never |
|  | Not counting carrots, potatoes, or salad, how many servings of other vegetables do you usually eat? | 0 to 99 [per day/per week/per month/per year], Never |
| Physical activity | Have you done any of the following in the past 3 months, that is, from [date three months ago] to yesterday? | No physical activity / 0 to 99 for [Walking for exercise / Gardening or yard work / Swimming / Bicycling / Popular or social dance / Home exercises / Ice hockey / Ice skating / In-line skating or rollerblading / Jogging or running / Golfing / Exercise class or aerobics / Downhill skiing or snowboarding / Bowling / Baseball or softball / Tennis / Weight-training / Fishing / Volleyball / Basketball / Soccer / Any other activity] |
|  | About how much time did you spend on each occasion? | 1 to 15 minutes, 16 to 30 minutes, 31 to 60 minutes, More than one hour |
